# Salicylic Acid Stimulates Antioxidant Defense and Osmolyte Metabolism to Alleviate Oxidative Stress in Watermelons under Excess Boron

**DOI:** 10.3390/plants9060724

**Published:** 2020-06-08

**Authors:** Mohamed Moustafa-Farag, Heba I. Mohamed, Ahmed Mahmoud, Amr Elkelish, Amarendra N. Misra, Kateta Malangisha Guy, Muhammad Kamran, Shaoying Ai, Mingfang Zhang

**Affiliations:** 1 Lab of Germplasm Improvement and Molecular Breeding, Agriculture and Biotechnology College, Zhejiang University, Hangzhou 310029, China; m_m_kamel2005@gdaas.cn (M.M.-F.); 11716103@zju.edu.cn (A.M.); malangisha@zju.edu.cn (K.M.G.); 2 Horticulture Research Institute, Agriculture Research Center, Giza 12619, Egypt; 3 Institute of Agricultural Resources and Environment, Guangdong Academy of Agricultural Sciences, Guangzhou 510640, China; drkamran2017@nwsuaf.edu.cn (M.K.); aishaoying@gdaas.cn (S.A.); 4 Biological and Geological Sciences Department, Faculty of Education, Ain Shams University, Cairo 11566, Egypt; hebaebrahem@edu.asu.edu.eg; 5 Botany Department, Faculty of Science, Suez Canal University, Ismailia 41522, Egypt; amr.elkelish@science.suez.edu.eg; 6 Department for Life Sciences, Central University of Jharkhand, Brambe, Ranchi-835205, India; anm@cuj.ac.in; 7 Department of Biosciences & Biotechnology, Khallikote University, GMax Building, Konishi, Berhampur 761008, India

**Keywords:** salicylic acid, chlorophyll fluorescence, excess boron, lipid peroxidation, enzymatic antioxidant, glutathione, proline, stomatal conductance

## Abstract

Boron (B) is a microelement required in vascular plants at a high concentration that produces excess boron and toxicity in many crops. B stress occurs widely and limits plant growth and crop productivity worldwide. Salicylic acid (SA) is an essential hormone in plants and is a phenolic compound. The goal of this work is to explore the role of SA in the alleviation of excess B (10 mg L^−1^) in watermelon plants at a morphological and biochemical level. Excess boron altered the nutrient concentrations and caused a significant reduction in morphological criteria; chlorophyll a, b, and carotenoids; net photosynthetic rate; and the stomatal conductance and transpiration rate of watermelon seedlings, while intercellular carbon dioxide (CO_2_) was significantly increased compared to the control plants (0.5 mg L^−1^ B). Furthermore, excess boron accelerated the generation of reactive oxygen species (ROS), such as hydrogen peroxide (H_2_O_2_) and induced cellular oxidative injury. The application of exogenous SA significantly increased chlorophyll and carotenoid contents in plants exposed to excess B (10 mg L^−1^), in line with the role of SA in alleviating chlorosis caused by B stress. Exogenously applied SA promoted photosynthesis and, consequently, biomass production in watermelon seedlings treated with a high level of B (10 mg L^−1^) by reducing B accumulation, lipid peroxidation, and the generation of H_2_O_2_, while significantly increasing levels of the most reactive ROS, OH^−^. SA also activated antioxidant enzymes, such as superoxide dismutase (SOD), peroxidase (POD), and ascorbate peroxidase (APX) and protected the seedlings from an ROS induced cellular burst. In conclusion, SA can be used to alleviate the adverse effects of excess boron.

## 1. Introduction

Boron (B) is an essential plant micronutrient and has a functional role in the creation and function of cell walls [1]. Boron stress causes drastic effects worldwide. Toxic concentrations of B occur in soils that are irrigated with water contaminated with B or an excessive use of B-rich fertilizer, sewage sludge, as well as natural deposits discovered throughout the globe in arid and semi-arid areas [2]. Boron stress induces certain biochemical and morphological failures, such as reduced shoot and root development [3,4]; photosynthesis inhibition; reduced stomatal conductance [5]; the generation of reactive oxygen species (ROS) causing oxidative stress to lipids, proteins, and nucleic acids [6]; reduced root proton extrusion [7], reduced root cell division [8]; lignin and suberin accumulation in roots [9]; increased permeability of the membrane; lipid peroxidation; and altered antioxidant enzyme activities [10]. Scientists have been interested in developing potential strategies to enhance the seeds germination and the plants growth to achieve higher crop production [11,12]. Using phytohormones such as salicylic acid (SA) can enhance plant tolerance and counteract the toxic impacts of heavy metals on the germination of seedlings and the growth and development of plants. SA is a natural plant hormone that acts as a signal molecule to regulate plant growth [11], seedling germination, glycolysis, the flowering process, fruit yield [11], the uptake and transport of ions [12], stomatal conductance, the transpiration and photosynthetic rate [13], and the regulation of antioxidant enzyme synthesis during both biotic and abiotic stress [14,15]. Additionally, SA is a crucial signal biomolecule mediated systemic resistance to pathogen attacks by plants [16]. SA has also been shown to protect winter wheat plants [17] against cold stress and heat stress [18,19] and to modulate plant responses to salt and osmotic stresses [15,20], ozone or UV light [21], drought [22], and heavy metals, such as Cd [23,24,25,26,27], Mn [28], Hg [29], B [30], and Pb [31].

Although SA is widely used to protect economic crops against abiotic stress, only a few reports investigated its protective impact under excess boron stress [32,33]. SA protects plants under excess boron associated salinity, such as spinach [34]. The protective role of SA is expressed mainly by regulating ROS and antioxidants, inducing gene expression, and absorbing and distributing elements [23,35,36]. The most effective effect of SA is to improve the function of antioxidant enzymes in vivo [37,38]. Although the role of SA in the antioxidant system is very well studied under different biotic and abiotic stresses [14,39], its role under B stress remains unclear. In addition, the relationship between SA and boron toxicity has had contrary results in different studies. For instance, in mung beans, SA inhibits lipid peroxidation and reduced boron toxicity disorders through a reduction in lipoxygenase activity [40]. Watermelon belongs to the Cucurbitaceous family and is considered one of the world’s top 20 cultivated crops with elevated economic value. The principal countries producing watermelon, such as China and Turkey [41,42], are affected by B stress, which has drastic effects on watermelon growth and yield. Nevertheless, there is insufficient knowledge on the mechanisms of boron toxicity-induced watermelons injury [4], highlighting the importance of alleviating B stress in watermelon crops.

Therefore, the aim of the present study is to (1) study the different responses of excess boron on watermelon and (2) evaluate the changes induced by SA in the antioxidant system and mineral uptake during excess boron stress. This research will help us to understand the mechanisms associated with excess boron in watermelon, help recommend the correct treatment of SA to alleviate B stress and aid the scientific community in solving the B stress problem.

## 2. Results

### 2.1. Plant Growth

The results show that the shoot and root dry weight of watermelon plants is significantly reduced by excess boron compared to control plants. In addition, the root projected area, surface area, diameter, volume, and number of tips was significantly decreased under excess boron except root volume, which experienced insignificant effects (Table 1). On the other hand, SA treatment (0.3 mM) increased watermelon growth and development under excess boron (Table 1).

### 2.2. Boron Uptake and Translocation

The accumulation of boron in the roots and leaves of watermelon was increased significantly by approximately 5.5- and 10-fold, respectively, in response to excess boron stress compared to the untreated plants (0.5 mg L^−1^) (Table 2). Watermelon plants translocated more B from the root to leaf tissues, as shown by the high translocation factor value (TF, the ratio of total B in the leaf and root tissues). The translocation factor showed a significant increase in excess boron treated plants. Treatment with SA caused a significant decrease in B contents in the roots and leaves of watermelon seedlings under excess boron stress compared to non-SA-treated plants.

### 2.3. Mineral Uptake

Without the SA treatment, a non-significant effect was found for the different macro-nutrients (Ca^2+^, Mg^2+^ and K^+^) in the shoots and root tissues under excess boron stress, but higher amounts of Na^+^ was recorded in the shoots of watermelon and lower amounts in roots under excess boron (Table 3). In the presence of SA, Ca^2+^ content was significantly reduced in shoots and a marked increase in roots compared to their respective non-SA plants under excess boron stress. Furthermore, treatment with SA caused a significant increase in the K^+^ content of watermelon shoots but decreased in roots under excess boron stress. SA-treated plants under excess boron stress had more Mg^2+^ in their root tissues, although the concentration in shoots remained unaffected. In addition, SA significantly decreased the shoot Na^+^ concentration in the boron-stressed plants and control plants. On the other side, the Na^+^ concentration increased dramatically in roots under excess boron and control conditions compared to non-SA plants (Table 3).

### 2.4. Chlorophyll and Carotenoid Content

**Figure 1:**
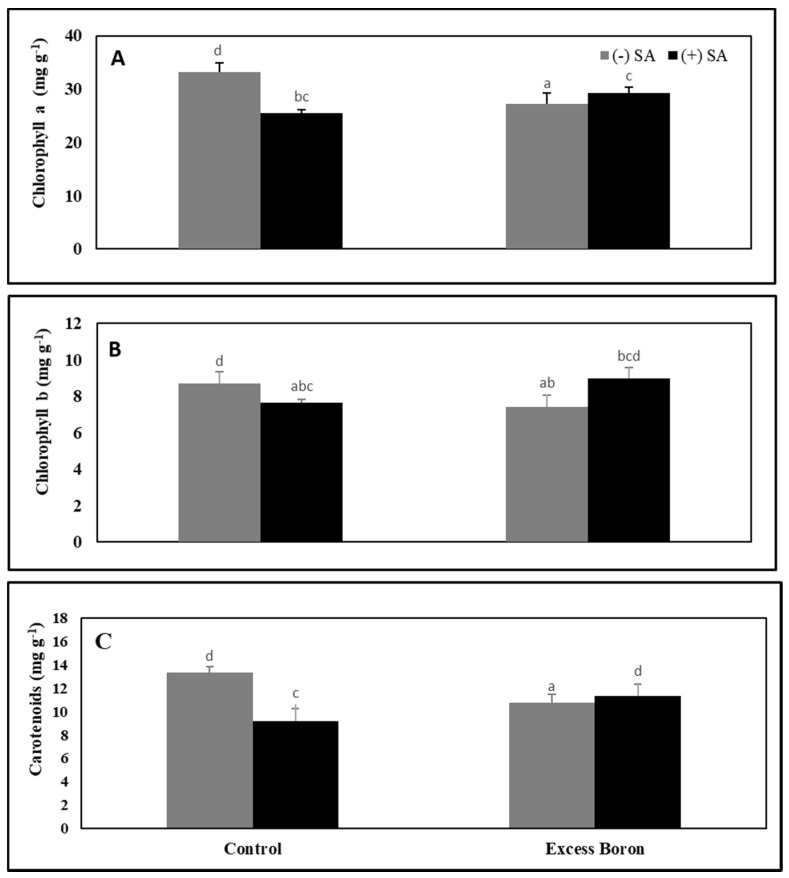
Effects of 0.3 mM SA on photosynthetic pigments (chlorophyll a (**A**), chlorophyll b (**B**) and carotenoids (**C**) in leaves under excess boron at 35 days old. The bars stand for mean ± SD. ANOVA were analyzed variations between the four treatments. The various letters indicate a significant difference from *p* < 0.05.

Excess boron stress caused a significant decrease in chlorophyll a, b, and carotenoid content in watermelon leaves compared to the untreated plants (Figure 1). The application of SA precipitated significant increases in chl a, chl b, and carotenoid content in stressed plants. These results confirm the role of SA in alleviating chlorosis symptoms caused by excess boron stress.

### 2.5. Leaf Gas Exchange

**Figure 2:**
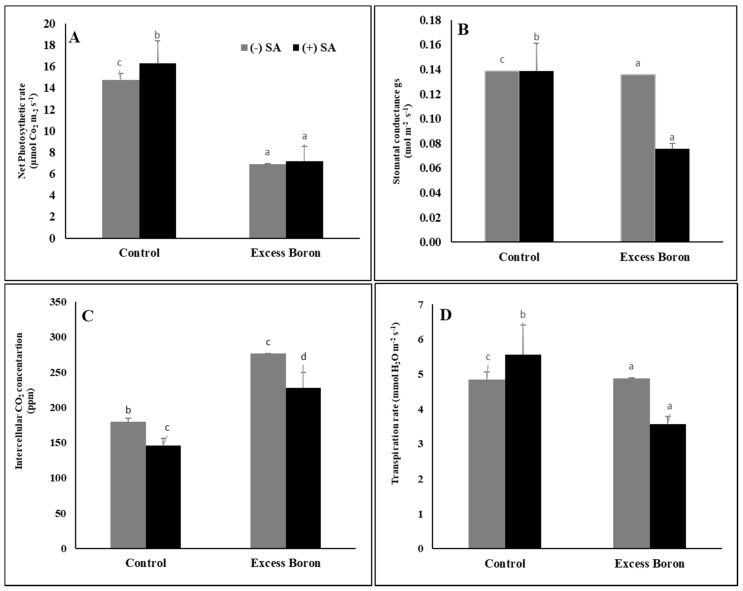
Effects of 0.3 mM SA on leaf gas exchange under excess boron at 35 days old. (**A**) Net photosynthesis rate, (**B**) stomatal conductance, (**C**) intercellular CO_2_ concentration, and (**D**) transpiration rate. Bars stand for mean ± SD. ANOVA were analyzed variations between the four treatments. The various letters indicate a significant difference from *p* < 0.05.

Excess boron exhibited significantly decreased PN, Gs, and Tr in watermelon seedlings and increased intercellular carbon dioxide (CO_2_) compared to the unstressed plants (Figure 2). In addition, treatment with SA induced a significant reduction in PN, Gs, Tr but a significant rise in intercellular carbon dioxide (CO_2_) under excess boron and in the control (Figure 2).

### 2.6. Chlorophyll Fluorescence

**Figure 3:**
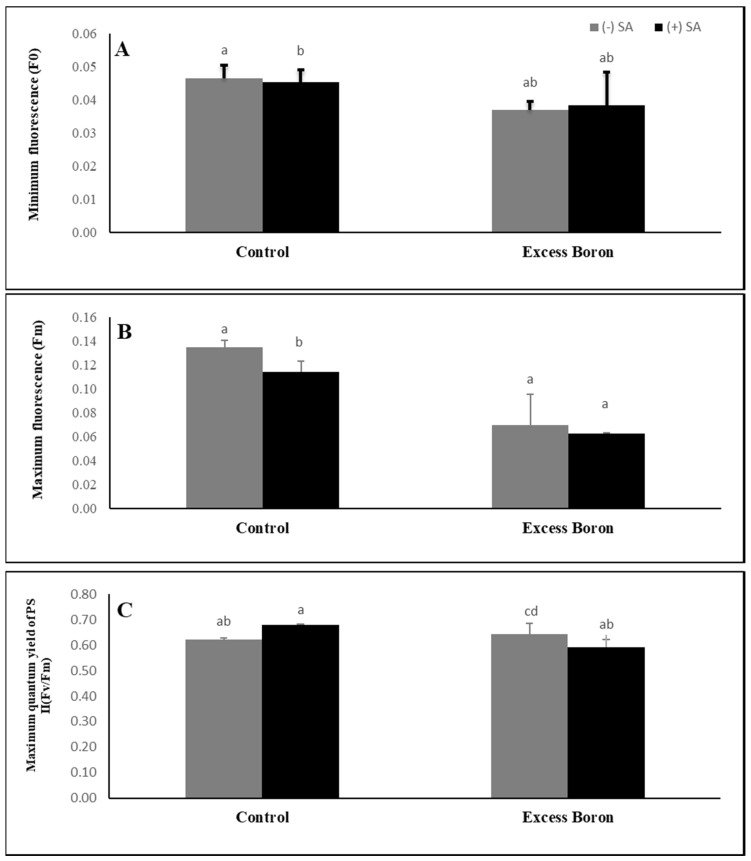
Effects of 0.3 mM SA on chlorophyll fluorescence under excess boron at 35 days old. (**A**) minimum fluorescence, (**B**) maximum fluorescence, and (**C**) maximum quantum yield. The bars stand for mean ± SD. ANOVA were analyzed variations between the four treatments. The various letters indicate a significant difference from *p* < 0.05.

Under excess boron stress, there are variable responses in the chlorophyll fluorescence of watermelon leaves (Figure 3). Excess boron induced a significant reduction in Fm but exerted a non-significant effect in F0 and PSII (Fv/Fm) compared to the control plants (0.5 B). Treatment with SA was significantly decreased in the PSII (Fv/Fm) of the plants under excess boron compared to the other treatments without SA (Figure 3), while F0 and Fm remained unaffected.

### 2.7. MDA Content and the Endogenous ROS Generation Rate

**Figure 4:**
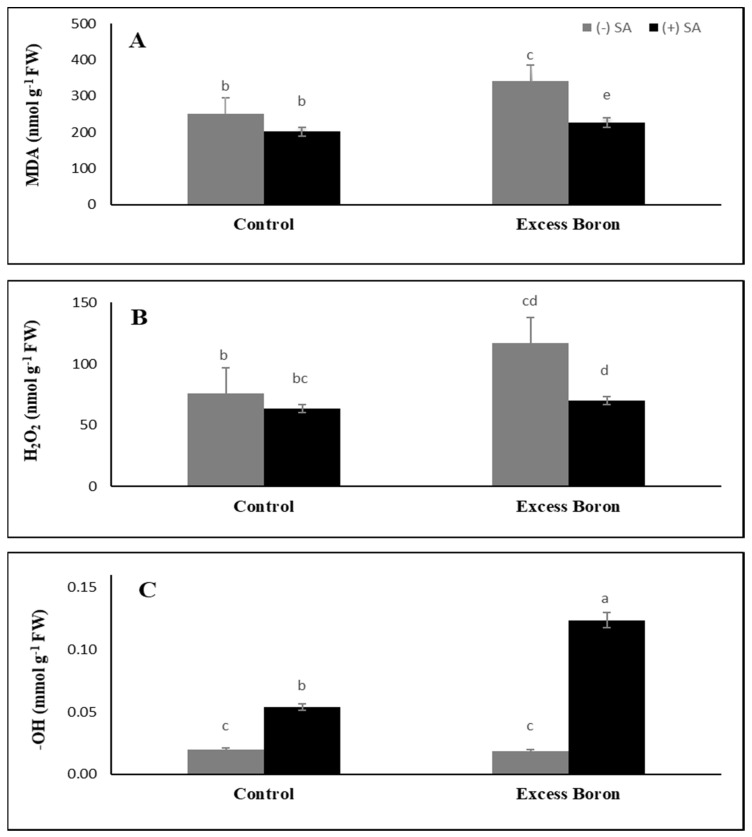
Effects of 0.3 mM SA on oxidative stress parameters under excess boron at 35 days old. (**A**) lipid peroxidation, (**B**) hydrogen peroxide, and (**C**) extra-cellular hydroxyl radicals. The bars stand for mean ± SD. ANOVA were analyzed variations between the four treatments. The various letters indicate a significant difference from *p* < 0.05.

A higher amount of the malondialdehyde (MDA) was recorded in boron-stressed plants as compared with the unstressed plants (Figure 4). Treatment with SA (0.3 mM) caused a significant decreased in the MDA content of watermelon leaves under control conditions and excess boron stress (Figure 4).

The generation of ROS such as H_2_O_2_ and OH^−^ formation was determined in B treated watermelon leaves (Figure 3). The production of ROS molecules in watermelon leaves has increased significantly by boron excess compared to the unstressed plants. Moreover, SA significantly reduced the generation of H_2_O_2_ in non-B-stressed plants and stressed plants. For instance, SA more effectively reduced H_2_O_2_ generation in excess boron plants (Figure 4).

### 2.8. Antioxidant Enzyme Activities

**Figure 5:**
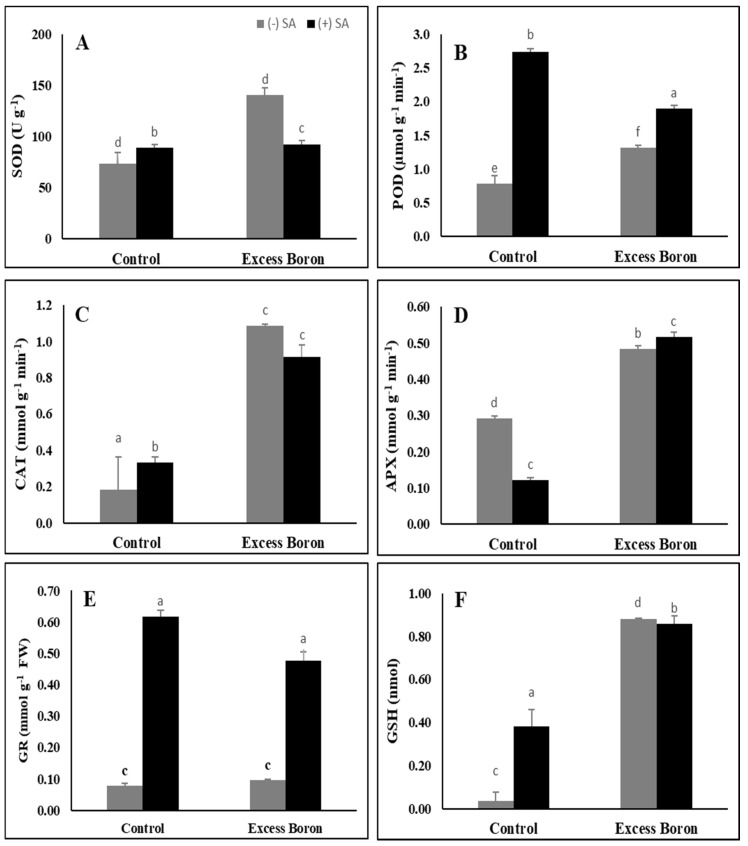
Effects of 0.3 mM SA on antioxidant enzyme activities under excess boron at 35 days old. (**A**) superoxide dismutase, (**B**) peroxidase, (**C**) catalase, (**D**) ascorbic peroxidase, (**E**) glutathione reductase, and (**F**) reduced glutathione. The bars stand for mean ± SD. ANOVA were analyzed variations between the four treatments. The various letters indicate a significant difference from *p* < 0.05.

The antioxidant enzymes activities (superoxide dismutase (SOD), ascorbate peroxidase (APX), peroxidase (POD), catalase (CAT), and glutathione reductase (GR) were significantly up-regulated in response to excess boron (Figure 5). The most pronounced accumulation (in POD, CAT, and APX) was detected in watermelon leaves under excess boron. SA treatments further improved the activities of POD, CAT, APX, and reduced glutathione (GSH) in the excess boron-stressed plants (Figure 5). In addition, SA significantly activated SOD, POD, CAT, and GSH in the control plants compared to their respective non-SA leaves (Figure 5).

### 2.9. Total Soluble Protein (TSP) and Proline Content

**Figure 6:**
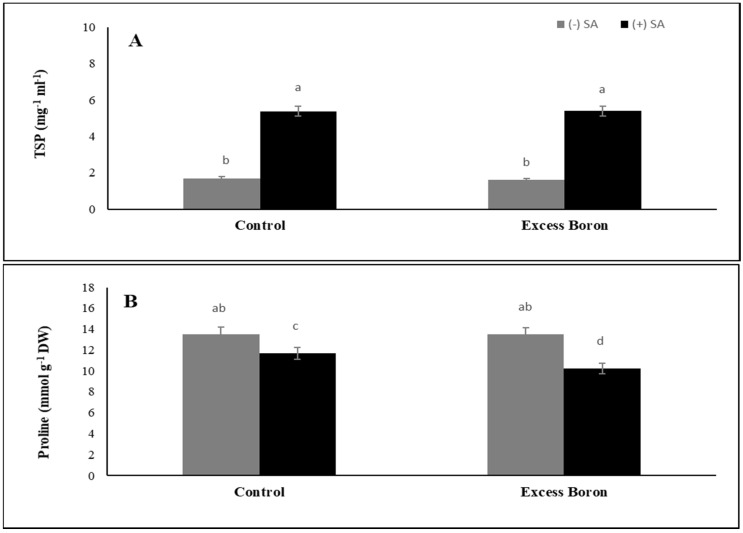
Effects of 0.3 mM SA on total soluble protein (**A**) and proline (**B**) content under excess boron at 35 days old. The bars stand for mean ± SD. ANOVA were analyzed variations between the four treatments. The various letters indicate a significant difference from *p* < 0.05.

TSP in the leaves of watermelon plants showed non-significant change under excess boron compared to the untreated plants (Figure 6). The application of SA exerted non-significant effects on the TSP in watermelon leaves under excess boron. On the other hand, the amount of proline in watermelon leaves was unaffected under excess boron, while the application of SA caused a significant inhibition of proline content in plants grown under excess boron (Figure 6).

## 3. Discussion

Boron is an important mineral nutrient needed for most plant species to progress and grow adequately. Excess boron can lead to lower growth, alter photosynthetic levels and cause morphological changes in the leaves [4]. In this experiment, we observed excess boron-induced chlorophyll and carotenoid deficiency in watermelon leaves. Analogous results were recorded by Aftab et al. [43]. The chlorophyll deficiency may be due to the production of degradation enzymes such as δ-aminolevulinic acid and protochlorophyllide under B stress [4]. Moreover, excess boron caused inhibition in plant growth (Table 1). The inhibition of growth in watermelon might result from the excess-boron-induced modification of essential metabolic processes, such as photosynthesis, and the nutrient uptake [4]. Furthermore, the inhibition of plant growth via excess boron may be due to the root cell division modulation and modification of the gene expression patterns associated with abscisic acid (ABA), or cell wall modifications, mitosis, water transport, and cell elongation [44,45].

The drop in photosynthetic pigments may be attributed to the accumulation of MDA and H_2_O_2_, resulting in the oxidation of chlorophyll and chloroplast membranes, which could be aggravated by excess B levels [4]. Treatment with SA protects photosynthetic pigments under excess boron. Some reports also indicate that SA is an effective photosynthesis regulator because it has a positive impact on the structure of the leaves and chloroplasts [46], as well as chlorophyll and carotenoid contents [47]. Alternatively, SA acts in the biogenesis for chloroplasts, protecting them against ROS and increasing chlorophyll stability [46]. In the present study, we observed an oxidative stress-induced reduction in the PN, Gs, and Tr of watermelon under excess boron stress (Figure 3). Similar results were reported by Lovatt et al. [5], who found that B reduced both Gs and the Pn transpiration rate.

Consequently, because of CO_2_ limits, PSII electrons are available from thylakoid and stromal fatty acids [48]. An SA supply significantly enhanced the Pn, Gs, and Tr of BD watermelon leaves compared to plants without an SA supply, which may prevent the oxidation of auxin, whose elevated contents increased Pn in the leaf [49]. Treatment with SA significantly decreased the PSII (Fv/Fm) of the plants under excess boron compared to their respective treatments without SA, suggesting that the exclusion of SA induced changes in chlorophyll fluorescence, from watermelon tolerance to B stress. This inhibition in the fluorescence of chlorophyll under excess boron may be due to oxidation of chlorophyll and chloroplast membranes, which may be exacerbated by excess B, as recorded in hot pepper [50].

SA participates in the regulation of multiple biochemical processes under excess boron stress. Our results showing excess boron are contrary to those in previous studies, which showed that SA decreased boron toxicity stress [32,33,40]. These differences may be due to the different excess boron and SA concentrations and genetic resources used in our study. Watermelon plants translocated relatively more B from the root to leaf tissues, as evidenced by the high value of the translocation factor (Table 2). Similarly, El-Feky et al. [32] found that the accumulation of B was significantly higher in barley shoots than in roots under boron toxicity [51]. SA not only reduced the stress conditions of BT by reducing the accumulation of B within the plant organs, but also by reducing the distribution of root to leaf B (Table 2). These results are in agreement with the findings of El-Feky et al. [32] and El-Feky et al. [32,52] on barley tissue. SA may work through certain specific processes, such as reducing the absorption or triggering the efflux from the roots, i.e., processes leading to lower cytoplasmic B content [30].

Unbalance in nutrients may result from excessive boron stress based on nutrient availability, absorption, transport and competition (Table 3). One mechanism for alleviating excess boron stress may be the shift of the mineral absorption after SA treatments. We postulate that SA lowered the damaging impacts of excess boron and enhanced the stability of the membrane and, therefore, the tolerance of the plant. In SA plants, the possible mechanism of B tolerance is the detoxification of excess B through cell exclusion and/or vacuolar compartmentalization. Furthermore, apoplast-formed B complexes could play a significant role in the excess boron tolerance of the plant. In this regard, Reid et al. [53] suggested that the transfer of B from the symplast into the apoplast or from the cytoplasm into the vacuole may facilitate plant tolerance to boron toxicity [54]. In plants, there are several reports that show excess boron stress-induced changes in the activity of both antioxidant enzymes and soluble antioxidant levels [3], accompanied by an enhancement of lipid peroxidation [55]. Excess boron can inhibit the transport of electrons and may result in the production and accumulation of ROS, such as H_2_O_2_ and OH^−^, in watermelon (Figure 4).

Moreover, SA application successfully inhibited the accumulation of MDA and the generation of H_2_O_2_ under excess boron stress compared to their respective SA alone. A high exogenous B supply could facilitate the transportation of boric acid to the cell, which could be partially converted to borate due to the cytosol’s elevated inner pH [56], thus potentially releasing free ROS, such as O_2_, OH, and H_2_O_2_ [8]. Similar findings were noted by [57], who clarified that MDA and H_2_O_2_ concentrations in safflower crops subjected to excess Zn are decreased significantly after supplementation with SA. It has been observed that SA relieves heavy metal-induced injury through metal chelation, scavenges lipid peroxyl radicals, and prevents lipid peroxidation through the activation of the antioxidant system and is, therefore, capable of protecting membrane integrity [58,59,60,61,62,63].

The protective effect of SA towards B stress strongly correlates with severalfold increases in levels of the hydroxyl radical (OH.), the most reactive ROS (Figure 4). SA can significantly elevate OH. levels even in unstressed (normal B) plants but OH. levels increased in SA-treated plants with B stress. It is known that OH. is mainly generated from H_2_O_2_ and Fe^2+^ via the Fenton reaction. The presence of free Fe is key to this process, e.g., in plants, where excess Fe is stored by ferritin proteins [64]. Therefore, it could be worth monitoring changes in Fe levels in SA-treated, B-stressed plants. Moreover, it is worth testing the effect of scavenging OH. and/or chelating Fe on the protective action of SA.

The activities of antioxidant defense enzymes, such as SOD, APX, POD, GR, and GSH, were significantly increased in response to B stress (Figure 5). These antioxidants play a critical role in the alleviation of abiotic stresses. For instance, SOD catalyzes the dismutation of superoxide to H_2_O_2_ and O_2_ as a significant scavenger. However, H_2_O_2_ is also poisonous to cells, and must be further drained by CAT or POD or both into water and O_2_ [65,66]. CAT and POD, when coordinated with SOD, seem to play an important protective role in scavenging ROS [67]. Our results are in agreement with those of Garcia et al. [68], who found that boron toxicity induced SOD activity in tobacco leaves. SA treatments further enhanced the activities of POD, CAT, and GR that were activated under excess boron conditions (Figure 5), suggesting that POD, CAT, and GR play a key role in the alleviation of both aspects of B stress. Similar results found that the application of SA activated antioxidant enzymes under zinc [57] and nickel stress [69].

Glutathione (GSH), a multi-functional plant metabolite, plays a significant role in oxidative stress in cell defense and safety. GSH protects thiol protein groups during stress from oxidation [70]. GSH content was significantly increased in watermelon plants under excess boron and SA treatments in stressed plants (Figure 5). Moreover, Cervilla et al. [2] found that GSH was significantly increased in only one tomato species under B stress. Yadav [71], likewise, suggested that the availability of GSH is related to the formation of phytochelatins, which are used to alleviate the toxicity of heavy metals. SA also function as an antioxidant [72], and the prevention of oxidative cell damage during stress has been suggested to be one of the stress tolerance mechanisms [73]. This level of safety was due to the increased production of antioxidants [18].

Proline content in watermelon plants showed a decrease under excess boron and control conditions after SA treatment (Figure 6). These findings are consistent with those of Namdjoyan et al. [57], who found that the addition of SA and sodium nitroprusside in Zn-stressed safflower crops caused a sharp decrease in proline content. SA’s function in proline degradation may be due to enhanced proline dehydrogenase activity [69].

The total soluble protein (TSP) in the leaves of watermelon plants experienced non-significant effects under excess boron and the application of SA but increased for the SA-treated plants compared to the non-stressed and stressed plants (Figure 6) [74,75]. SA induced a considerable increase in the content of protein fractions in sunflower plants under Cu stress [76] and chamomile plants under Cd and Ni stresses [77].

## 4. Materials and Methods

### 4.1. Plant Materials, Growth Conditions, and Treatments

Watermelon seeds are sterilized and then soaked in distilled water for one day at room temperature. Then, the seeds are germinated for 5 days in the dark on filter papers. The seedlings were transplanted into 5 L plastic boxes containing full Hoagland, while excess boron stress was applied by adding boric acid (H_3_BO_3_) into the solution at 10 mg L^−1^. The concentrations of B were chosen based on preliminary studies using different concentrations of B to choose the suitable concentrations for excess boron and according to the literature [3,4]. SA (0.3 mM) supplementations were added to the Hoagland solution directly after transplanting into the plastic boxes. In our experiments, we used 2 concentrations of B and 2 concentrations of SA (4 treatments). Each treatment used 30 plants divided into three boxes (10 plants for each box). The plants grew for 30 days in growth chambers under 20–25/15–20 °C (day/night) and 60% humidity. The solutions were changed twice a week, and the pH of the solution was preserved between at 6.0 and 6.3.

Leaves from the third middle part were used for photosynthetic gas exchange and biochemical aspects.

### 4.2. Morphological Measurements

Following 1 month of the treatments, 10 seedlings were harvested from each treatment. The plants were dried out at 80 °C for 3 days. The root system, including surface areas, root volume, root diameter, and the number of root tips, was analyzed with WinRhizo Pro (S) v. 2009a software, in accordance with Yu et al. [78].

### 4.3. Determination of B and Uptake of Other Nutrients

The dried shoot and root samples were digested in a mixture of nitric acid and hydrogen peroxide at 130 °C. Mineral concentrations of B, Ca^2+^, Mg^2+^, K^+^, and Na^+^ were assayed by coupled plasma mass spectrometry (ICP-MS, Agilent 7500a, Agilent Technologies, Santa Clara, CA, USA) [79].

### 4.4. Malondialdehyde (MDA) and Reactive Oxygen Species (ROS)

Lipid peroxidation was measured by malondialdehyde (MDA), according to Zhou et al. [80].

Hydrogen peroxide was measured via the method described by Yordanov et al. [81] with a slight modification. The absorbance was read at 630 nm after 15 min.

Extra-cellular hydroxyl radicals (OHs) from the leaf samples were estimated following the protocol of Halliwell et al. [82]. The reaction mixtures contained, in a final volume of 1.0 mL, the following reagents at the following concentrations: deoxyribose (variable concentration), KH_2_PO_4_-KOH buffer, pH 7.4 (20 mM), FeCl_3_ (100 µM), EDTA (104 µM), H_2_O_2_ (1 mM), and ascorbate (100 µM). Solutions of FeC1_3_ and ascorbate were produced immediately before use in deaerated water. Reaction mixtures were incubated at 37 °C for 1 h, and the color was developed at 532 nm using a spectrophotometer.

### 4.5. Chlorophyll and Carotenoid Determination

Fresh leaf tissues were ground in the presence of liquid nitrogen. Methanol (95%) was added to the samples, and the mixture was centrifuged for 5 min at 4500 rpm at 20 °C. The absorbance of the extracted solution was recorded at wavelengths of 470, 649 and 665 nm to estimate the chlorophyll-a, chlorophyll-b [83], and carotenoids [84] using a spectrophotometer (Shimadzu UV-1700, Tokyo, Japan).

### 4.6. Photosynthetic Gas Exchange Parameters

The photosynthetic rate (Pn), stomatal conductance (Gs), transpiration rate (Tr), and intercellular CO_2_ were recorded using a portable LI-COR 6400 photosynthesis system (Lincoln, NE, USA). The intrinsic water use efficiency (WUE) was calculated from the ratio of the photosynthesis rate and stomatal conductance [84].

### 4.7. Chlorophyll Fluorescence

The maximal quantum yield of PSII (Fv/Fm), initial fluorescence (F0), and maximum fluorescence (Fm) were determined by an imaging pulse amplitude modulation (PAM) device (IMAG-MAXI; Heinz Walz, Effeltrich, Germany).

### 4.8. Antioxidant Enzymes

Fresh leaf samples (0.5 g) (4 replicates in total) were homogenized in cold phosphate buffer (pH 7.8). The homogenate was centrifuged at 13,000× *g* for 20 min at 4 °C, and the supernatant was used to determine the enzymatic activities.

Superoxide dismutase activity (SOD: EC 1.15.1.1) was determined following the method of Zhang et al. [85]. One unit of SOD was defined as the amount contained in the volume of extract that caused a 50% inhibition of the SOD-inhibitable fraction of the Nitro blue tetrazolium (NBT) reduction.

Peroxidase activity (POD: EC 1.11.1.7) was assayed according to Velikova et al. [86]. The absorbance was recorded at 470 nm.

Catalase activity (CAT: EC 1.11.1.6) was measured according to Aebi [87]. The consumption of H_2_O_2_ was monitored using a spectrophotometer at 240 nm (e = 39.4 mM^−1^·cm^−1^) for 3 min.

Ascorbate peroxidase activity (APX: EC 1.11.1.11) was estimated by the method of Nakano et al. [88]. The absorbance was read at 265 nm (e = 13.7 mM^−1^·cm^−1^).

Glutathione reductase (GR: EC 1.6.4.2) activity was assayed according to Jiang et al. [89] after the oxidation of NADPH at 340 nm (with an extinction coefficient of 6.2 mM·cm^−1^) for 1 min. Reduced glutathione (GSH) was determined according to Law et al. [90]. An increase in the absorbance at 412 nm was measured after the addition of 5,5-dithiobis (2-nitrobenzoic acid) (DTNB).

### 4.9. Total Soluble Protein and Proline

Total soluble protein (TSP) was determined according to the Bradford [91] method using bovine serum albumin as a standard. For proline determination, sulphosalicylic acid methods are being applied and the absorbance was recorded at 520 nm using a spectrophotometer [92].

### 4.10. Statistical Analysis

A completely randomized block design was used in our study, and the biostatistics were analyzed using CoStatv. 6.4 software. The results are denoted as the mean ± SD.

## 5. Conclusions

The present work demonstrates that SA can alleviate excess boron. The mechanisms by which SA alleviates excess boron mainly involve the following activities: (1) promoting chlorophyll content, (2) modulating the balance of mineral elements, (3) protecting against oxidative stress by decreasing MDA and ROS production, and (4) acting directly as an antioxidant to scavenge reactive oxygen species and/or indirectly modulating redox balance through the activation of the antioxidant responses. Our results could facilitate an integrated and optimized management strategy to alleviate excess boron in watermelon, but further study is needed.

## Figures and Tables

**Table 1:** Effect of 0.3 mM salicylic acid (SA) on watermelon root system development under excess boron at 35 days old.

Treatment	Shoot Dry Weight	Root Dry Weight	Project Area (cm^2^)	Surface Area (cm^2^)	Avg Diameter (mm)	Root Volume (cm^3^)	Number of Tips
**Control**	7.0 ± 0.42 ^a^	0.17 ± 0.010 ^b^	42.85 ± 2.57 ^b^	90 ± 5.4 ^c^	0.64 ± 0.04 ^d^	1.48 ± 0.09 ^b^	3173 ± 190 ^b^
**Excess B**	1.54 ± 0.09 ^d^	0.11 ± 0.007 ^e^	20.25 ± 1.21 ^e^	59 ± 3.6 ^e^	0.72 ± 0.04 ^c^	1.23 ± 0.07 ^c^	1161 ± 69 ^e^
**Control + SA**	3.78 ± 0.29 ^b^	0.29 ± 0.022 ^a^	51.16 ± 3.99 ^a^	160 ± 12.5 ^a^	0.80 ± 0.06 ^b^	3.20 ± 0.25 ^a^	4162 ± 324 ^a^
**Excess B + SA**	2.29 ± 0.22 ^c^	0.14 ± 0.014 ^d^	24.03 ± 2.40 ^d^	73 ± 7.4 ^d^	0.49 ± 0.05 ^e^	1.54 ± 0.15 ^b^	1921 ± 192 ^d^

Columns stand for mean ± SD. ANOVA were analyzed variations between the four treatments. Various letters indicate a significant difference from *p* < 0.05.

**Table 2:** Effect of 0.3 mM SA on boron (B) uptake and translocation on watermelon leaves and roots under excess boron at 35 days old.

Treatment	B Level in Roots (mg kg^−1^)	B Level in Leaves (mg kg^−1^)	Translocation Factor
**Control**	73.9 ± 3.05 ^c,d^	150.9 ± 20 ^c^	2.04 ± 0.35 ^b^
**Excess B**	408.0 ± 40 ^a^	1592.3 ± 400 ^a^	3.92 ± 1.36 ^a^
**Control + SA**	80.3 ± 6 ^c^	90.2 ± 6 ^c^	1.12 ± 0.07 ^c^
**Excess B + SA**	276.3 ± 40 ^b^	1146.3 ± 200 ^b^	4.18 ± 1.33 ^a^

Columns stand for mean ± SD. ANOVA were analyzed variations between the four treatments. Various letters indicate a significant difference from *p* < 0.05.

**Table 3:** Effect of 0.3 mM SA on nutrient uptake and translocation in watermelon shoot and root tissues under excess boron at 35 days old.

Treatment	Macro-Nutrient (mg kg^−1^ Shoot DW)			
	Ca^2+^	K^+^	Mg^2+^	Na^+^
**Control**	60.8 ± 5.03 ^a^	87.5 ± 4 ^b,c^	16.1 ± 2 ^a^	15.1 ± 2 ^c^
**Excess B**	56.9 ± 4 ^a,b^	79.8 ± 4 ^c^	14.0 ± 2 ^a^	19.0 ± 2 ^b^
**Control + SA**	54.6 ± 4 ^b^	103.7 ± 20 ^a^	14.1 ± 2 ^a^	1.2 ± 0.2 ^d^
**Excess B + SA**	49.8 ± 4 ^c^	96.7 ± 12 ^a,b^	15.4 ± 4 ^a^	1.7 ± 0.2 ^d^
**Treatment**	**Macro-Nutrient (mg kg^−1^ Root DW)**			
	**Ca^2+^**	**K^+^**	**Mg^2+^**	**Na^+^**
**Control**	12.4 ± 4 ^c^	107.5 ± 20 ^a^	3.4 ± 0.4 ^c^	4.2 ± 0.4 ^b^
**Excess B**	14.5 ± 4 ^c^	112.8 ± 20 ^a^	3.5 ± 0.4 ^c^	3.4 ± 0.4 ^e^
**Control + SA**	24.8 ± 2 ^b^	68.2 ± 4 ^b^	6.0 ± 0.2 ^a^	8.9 ± 0.2 ^a^
**Excess B + SA**	22.7 ± 2 ^b^	34.8 ± 4 ^c^	4.9 ± 0.2 ^b^	4.2 ± 0.2 ^b^

Columns stand for mean ± SD. ANOVA were analyzed variations between the four treatments. Various letters indicate a significant difference from *p* < 0.05.
